# Epigenetic dysregulation and biological function of PDX1 in prostate cancer

**DOI:** 10.18632/oncotarget.28854

**Published:** 2026-03-31

**Authors:** Tayo A. Adeyika, Anju Datturgi, Yehnara Ettinoffe, Somiranjan Ghosh, Christopher Albanese, Bernard Kwabi-Addo

**Affiliations:** ^1^Department of Biochemistry and Molecular Biology, Howard University, Washington, DC 20059, USA; ^2^Department of Pharmaceutical Sciences, Howard University, Washington, DC 20059, USA; ^3^Department of Oncology, Lombardi Comprehensive Cancer Center, Georgetown University Medical Center, Washington, DC 20057, USA; ^4^Department of Biology, Howard University, Washington, DC 20059, USA; ^5^Department of Pediatrics and Child Health, College of Medicine, Howard University, Washington, DC 20059, USA; ^6^Howard University Cancer Center, Howard University, Washington, DC 20059, USA

**Keywords:** PDX1, DNA methylation prostate cancer, shRNA knockdown, over-expression, glucose

## Abstract

Aberrant DNA methylation changes lead to abnormal gene expression that contributes to the development and progression of prostate cancer (PCa). Inquiry of genome-wide DNA methylation dataset, we identified the homeodomain pancreatic and duodenal homeobox 1 (*PDX1*) gene as differentially hypermethylated in PCa compared to normal prostate tissues. Immunohistochemical analysis of matched PCa and normal prostate tissues using tissue microarray showed a significant 2.33-fold (*p* = 0.0001) higher PDX1 protein expression in the PCa compared to the normal prostate tissues. In PCa cell lines (PC-3 and LNCaP) engineered to stably overexpress or knockdown PDX1, the ectopic PDX1 expression significantly enhanced cell proliferation and migration, whereas PDX1 knockdown suppressed these phenotypic processes. Quantitative RT-PCR and Western blot analysis demonstrated that PDX1 overexpression was associated with increased expression of key metabolic regulators; INSR, IGF1R, CXCR4, CDH2, TWIST1, and SNAI1, whereas there is decreased expression of ESR2, and TNFα. Conversely, PDX1 knockdown led to the opposite effect in expression profiles of these metabolites. Notably, these effects were more pronounced under high-glucose conditions compared to low-glucose environments. Overall, our findings suggest that PDX1 plays a tumor-promoting role in human PCa cells by influencing expression of metabolites in insulin, inflammatory, and epithelial-mesenchymal transition (EMT) signaling pathways. Given its potential role in metabolic regulation, full insights into the function of PDX1 in PCa could contribute to improved treatment and prevention strategies, particularly for men with PCa and comorbidities such as obesity and diabetes.

## INTRODUCTION

Prostate cancer (PCa) is the most common cancer in men, characterized by its heterogeneity, ranging from slow-growing, indolent cases to aggressive forms with fatal outcomes [1]. In 2024, the American Cancer Society estimated that 299,010 men would be newly diagnosed with PCa, with 35,250 deaths attributed to the disease [2]. Three well-established risk factors for prostate cancer include age, race, and family history [3]. Age is the most significant yet least understood risk factor, as PCa is rarely diagnosed in men under 50 years old (<0.1% of cases). The average age at diagnosis is between 72 and 74 years, with approximately 85% of cases occurring in men over 65 [4]. Racial disparities in PCa incidence and mortality are evident, with African American (AA) men having a higher likelihood of diagnosis, greater chances of presenting with distant metastases, and nearly 2.5 times the mortality rate compared to European American (EA) men [5]. Additionally, a family history of PCa is a well-recognized risk factor, reflecting a complex interplay of genetic and environmental influences [6, 7].

Environmental factors, particularly diet and lifestyle, play a significant role in PCa risk. Historically, PCa incidence and mortality have been higher in the United States and Western Europe compared to Asia and Africa [8]. However, second- and third-generation Asian migrants to the U.S. who adopt a Western diet experience a significant increased risk of PCa, comparable to European American (EA) men and markedly higher than their counterparts in Asian countries [9]. This pattern strongly suggests that environmental exposures, including diet, influence PCa initiation and progression or that conditions in less developed countries may inhibit prostatic carcinogenesis [9]. Specifically, high intake of animal fat from red meat has been consistently linked to an increased risk of PCa, whereas the consumption of tomatoes, soy, and other vegetables may offer protective benefits [10–12].

Obesity influences the entire PCa continuum, from detection and diagnosis to treatment and survivorship. It is widely recognized that obesity is linked to an increased risk of aggressive PCa [13]. Additionally, obesity is associated with larger prostate volume and significantly lower prostate-specific antigen (PSA) levels, which may contribute to delays in PSA-based prostate cancer diagnosis [14]. A review by Yang et al. highlights the growing cancer burden attributed to adiposity throughout life, supported by epidemiological associations between obesity, metabolic syndrome, increased PCa incidence, higher biochemical recurrence rates, and elevated PCa-specific mortality [15]. Stratified meta-analyses consistently demonstrate a strong correlation between body mass index (BMI) and increased PCa mortality [16]. Obese men with diabetes face an even greater risk of aggressive PCa, as both conditions contribute to disease progression [17, 18]. Several metabolic disorders, including hyperglycemia, hyperinsulinemia, and increased levels of proinflammatory metabolites, may further influence PCa risk in individuals with obesity, regardless of diabetes status. Obesity is characterized by low-grade chronic inflammation, driven by fatty acids, inflammatory cytokine production, and immune cell infiltration, all of which promote the release of inflammatory mediators [19]. These mediators include cytokines (interleukins and TNFs) and chemokines (CC, CXC, XCL, and C-X3-C family members), which play crucial roles in immune responses, inflammation, tumor development, and metastasis [20, 21]. Moreover, single nucleotide polymorphisms (SNPs) in various inflammatory genes can alter gene expression, influencing the development and progression of multiple cancers, including PCa [22].

However, the molecular mechanisms linking obesity to increased PCa mortality remain largely unclear. It is uncertain whether carcinogenesis in obese men leads to more aggressive cancer from the outset or if early carcinogenesis occurs independently of obesity, with obesity subsequently accelerating neoplastic progression. One study suggests that obesity is associated with poorer PCa prognosis, particularly in men whose tumors harbor the TMPRSS2: ERG gene fusion [23], providing a potential molecular link between obesity and PCa outcomes. Obesity disrupts multiple biological pathways that may influence PCa progression, including alterations in circulating levels of insulin, free insulin-like growth factor 1 (IGF-1), adiponectin, and sex hormones when compared to normal-weight men [24].

Recent epigenome-wide studies have established associations between adiposity, diabetes, and obesity-related chronic diseases [25–27]. However, few studies have explored the epigenetic dysregulation of metabolic markers and their relationship with obesity, PCa, and disease disparities. In our preliminary genome-wide DNA methylation analysis, we identified differential hypermethylation of the *Pancreatic and Duodenal Homeobox 1* (*PDX1*) gene, in high-grade PCa compared to normal tissues, that is consistent with *PDX1* DNA methylation pattern in the TCGA dataset [28]. The *PDX1* gene encodes a transcription factor essential for early pancreatic differentiation and is highly expressed in pancreatic beta cells (β-cells), where it plays a key role in regulating insulin gene expression [29]. Epigenetic regulation of *PDX1* has been implicated in pancreatic tissues as a potential mechanism in obesity-related type 2 diabetes mellitus (T2DM) development [30], while dysregulation of homeodomain transcription factors has been linked to tumor progression and metastasis [31]. The precise role of PDX1 in PCa remains unclear. Given the established link between obesity, diabetes, and increased PCa risk in AA men, aberrant *PDX1* epigenetic modifications may serve as a potential mechanism underlying aggressive prostate carcinogenesis in this population. To further investigate this, the present study aims to examine the role of PDX1 signaling and glucose homeostasis in PCa cell lines, providing insights into its potential contribution to prostate tumor progression.

## RESULTS

### Comparative analysis of PDX1 genome-wide DNA methylation and protein expression

**Figure 1:**
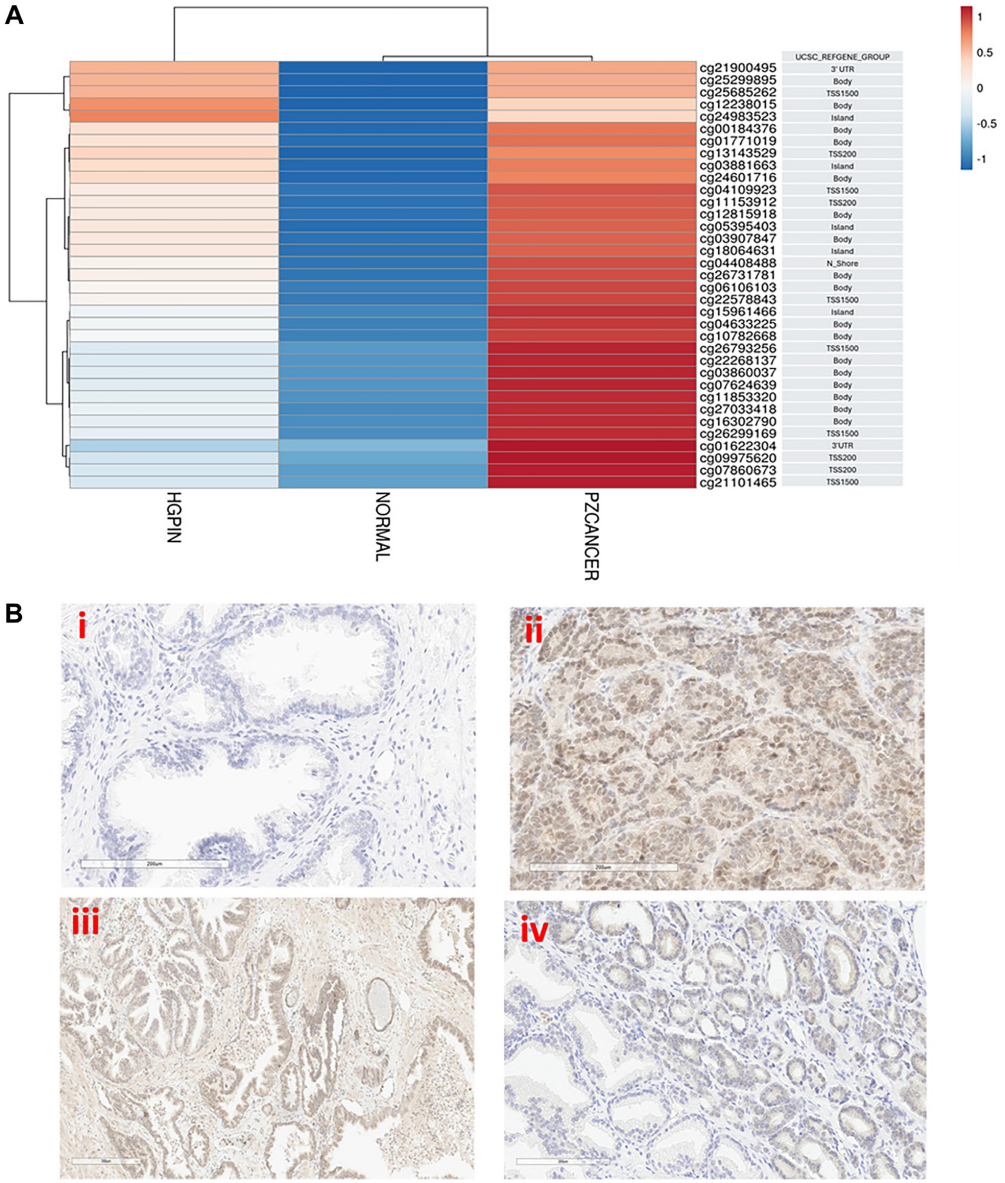
(**A**) DNA methylation and immunohistochemical analysis of PDX1 in prostate tissues. (A) Heat map analysis compares the methylation level of unique CpG probe-sets of PDX1 genome in Illumina 450K genome-wide DNA methylation array dataset in pre-cancerous lesion HGPIN, Normal and Prostate Cancer (PZCancer) tissues. Data shows beta (β)-values expressed as log_10_β-value (FDR adjusted; *p* < 0.05). Where adjusted β-values <−0.5 indicates hypomethylation and β-values >0.5 indicates hypermethylation. The UCSC_REFGENE_GROUP shows the genomic region of each PDX1 CpG probe-set analyzed. (**B**) Immunohistochemical analysis of PDX1 expression in tissue microarrays. Expression of PDX1 in normal prostate (**i**) and prostate cancer (**ii**–**iv**) was determined using tissue microarrays as described in “Materials and Methods.” (i) normal prostatic epithelial cells with no PDX1 expression. (ii) prostate cancer epithelial cells with both nuclear cytoplasmic strong expression of PDX1. (iii) Heterogenous PDX1 expression in the prostate tumors. (iv) Prostate tumors showing both positive and negative PDX1 expression. The mean staining intensity for normal (0.96 ± 0.02) and PCa (2.24 ± 0.44); where PCa staining intensity is significantly higher in PCa compared to matched normal tissues (*p* < 0.0001).

To explore differential methylated CpG sites in PDX1 genome between PCa and normal prostate tissues, we analyzed all CpG probe-sets designed for PDX1 genome in our previously published genome-wide DNA methylation dataset [32]. Heatmap cluster analysis of the methylation status measured as beta-value (FDR adjusted; *p*-value <0.05) showed hypomethylation of most probesets in the normal prostate tissues, intermediate methylation level in HGPIN and hypermethylation in the PCa (PZCancer, Figure 1A). To ascertain the expression of PDX1 in PCa, we analyzed a total of 25 prostate cancers and matched normal tissue microarrays by immunohistochemistry. The tissue microarrays contain 5-μ tissue cores from cancers in triplicate as well as normal tissue cores (also in triplicate) from patients undergoing radical prostatectomy. The overall expression of PDX1 is quite heterogenous in both cancer and adjacent normal tissues (Figure 1B). In 3 normal cases, there was no expression (data not shown), whereas vast majority of normal cases showed low expression (Figure 1B (i)). The vast majority of cancer cases showed variable PDX1 expression and mostly moderate to strong expression of PDX1 (Figure 1B (ii, iii)). PDX1 is predominantly expressed in the cytoplasm in most cases, although there are few cases of both cytoplasmic and nuclear expression (Figure 1B (ii)) and some cases that showed both positive and negative PDX1 expression (Figure 1B (iv)). A semi-quantification of PDX1 expression was calculated based on staining intensity (determined by intensity of staining as well as its pattern of localization). The overall PDX1 staining intensity was significantly higher in PCa (2.33-fold; *p* < 0.0001; student *T*-test) compared to normal tissues. Therefore, although PDX1 is hypermethylated in PCa than normal tissues, the expression level is also higher in PCa than normal tissues. Our observation is consistent with the large TCGA dataset; whereby hypermethylation of PDX1 positively corelated with high transcriptomic expression in prostate adenocarcinoma compared with normal prostate tissues [33].

### Functional validation of PDX1 expression on PCa cell proliferation and migration

**Figure 2:**
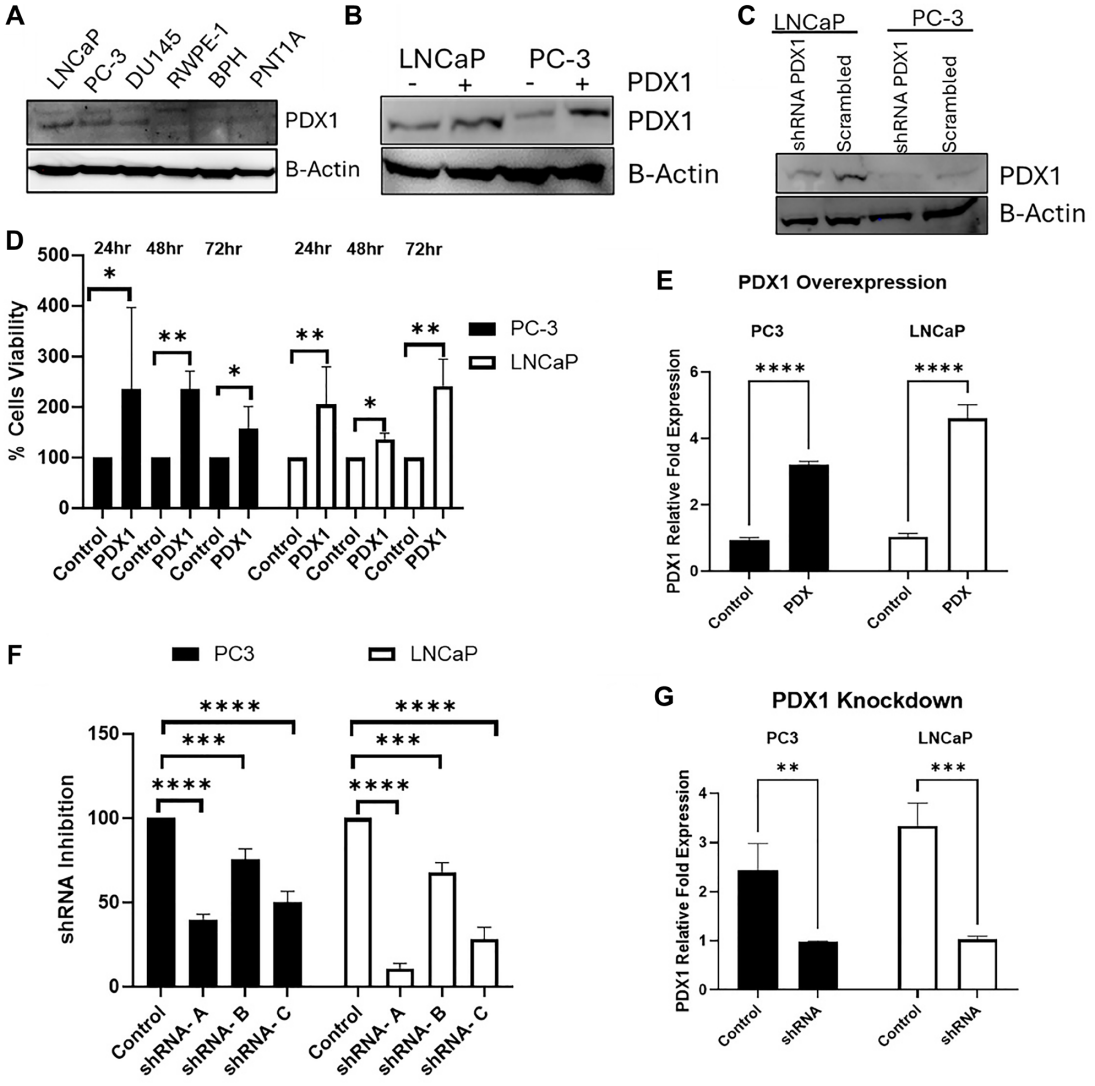
PDX1 expression and functional impact on prostate cancer cell viability. (**A**) Western blot analysis of basal expression levels of PDX1 in PCa cell lines (androgen-dependent LNCaP cells, and androgen-independent PC-3 and DU145 cells); primary immortalized epithelial cell lines (RWPE1 and pNT1A cells) and Benign Prostatic Hyperplasia (BPH) cell lines. (**B**) Western blot analysis of PDX1 expression in LNCaP and PC-3 cells stably transfected with either control (−) or PDX1 expression vector (+). (**C**) Western blot analysis of PDX1 expression in LNCaP and PC-3 cells stably transfected with either PDX1 shRNA vector or scramble shRNA control. (**D**) Cell viability assay showing the relative % cell viability effect of PDX1 expression in LNCaP and PC-3 cells, compared to control at different time points (24, 48, and 72 hours). (**E**) QRT-PCR analysis of PDX1 expression in stably transfected PC-3 and LNCaP cells compared to control. (**F**) Percentage inhibition of cell viability in LNCaP and PC-3 cells stably transfected with different PDX1 shRNA compared to scrambled shRNA transfections. (**G**) QRT-PCR analysis of the relative expression of PDX1 shRNA knockdown in PC-3 and LNCaP cells compared to scrambled shRNA control. Controls are set to 1 for (E) and (G); and controls are set to 100% for (D) and (F). Data shown are representative of three independent experiments. Statistical significance is indicated as (^*^p < 0.05; *t*-test).

To assess the functional consequences of *PDX1* gain or loss of expression, we conducted experiments in androgen-dependent LNCaP and androgen-independent PC-3 PCa cell lines. Baseline *PDX1* protein expression as revealed by Western blot analysis in a panel of six prostate cell lines (Figure 2A) showed heterogeneous PDX1 expressions, with high levels in LNCaP, moderate levels in PC-3 and DU145, and low levels in RWPE1, BPH, and pNT1A cells. Stable PDX1-overexpressing LNCaP and PC-3 cell lines were generated by transiently transfecting cells with pCMV-*PDX1* (encoding the full-length *PDX1* open reading frame) or an empty pCMV vector as control (all vector only transfections are hereafter referred to as control), followed by selection in neomycin-containing media until isogenic clones were established. Western blot analysis demonstrated that LNCaP and PC3 cells stably transfected with the *PDX1* gene, showed higher PDX1 protein expression compared to the control only transfection (Figure 2B). Conversely, stably engineered PDX1 knockdown was achieved using a PDX1 shRNA lentiviral vector or a scrambled shRNA control, with selection in puromycin-containing media until isogenic clones were established. Western blot analysis demonstrates reduced PDX1 expression in the PDX1 knockdown cells compared to the scrambled control cells (Figure 2C). Compared to controls, PDX1 overexpression significantly enhanced cell proliferation over a 72-hour period in both LNCaP and PC-3 cell lines (Figure 2D). Specifically, PC-3 cells exhibited a relative 2.3-fold increase in proliferation at 48 hours post-transfection, while LNCaP cells showed a relative 2.4-fold increase at 72 hours post-transfection (Figure 2D).

Quantitative RT-PCR analysis revealed significant 3.2-fold increase in PDX1 transcript expression in PC-3 cells and a 5.4-fold increase in LNCaP cells compared to control transfections (Figure 2E). In contrast, PDX1 knockdown using PDX1 shRNA led to a significant reduction in cell proliferation compared to the scrambled shRNA control. The three PDX1 shRNAs resulted in inhibition ranging from 1.32-fold to 2.52-fold in PC-3 cells and from 3.55-fold to 14.8-fold in LNCaP cells (Figure 2F). For the PDX1 shRNA that showed the greatest inhibition, quantitative RT-PCR analysis demonstrated a 2.4-fold and 3.5-fold reduction in PDX1 transcript expression in PC-3 and LNCaP cells, respectively, compared to the scrambled shRNA controls (Figure 2G). These gain-and-loss functional analyses highlight a tumor-promoting role of PDX1 in both LNCaP and PC-3 cells.

**Figure 3:**
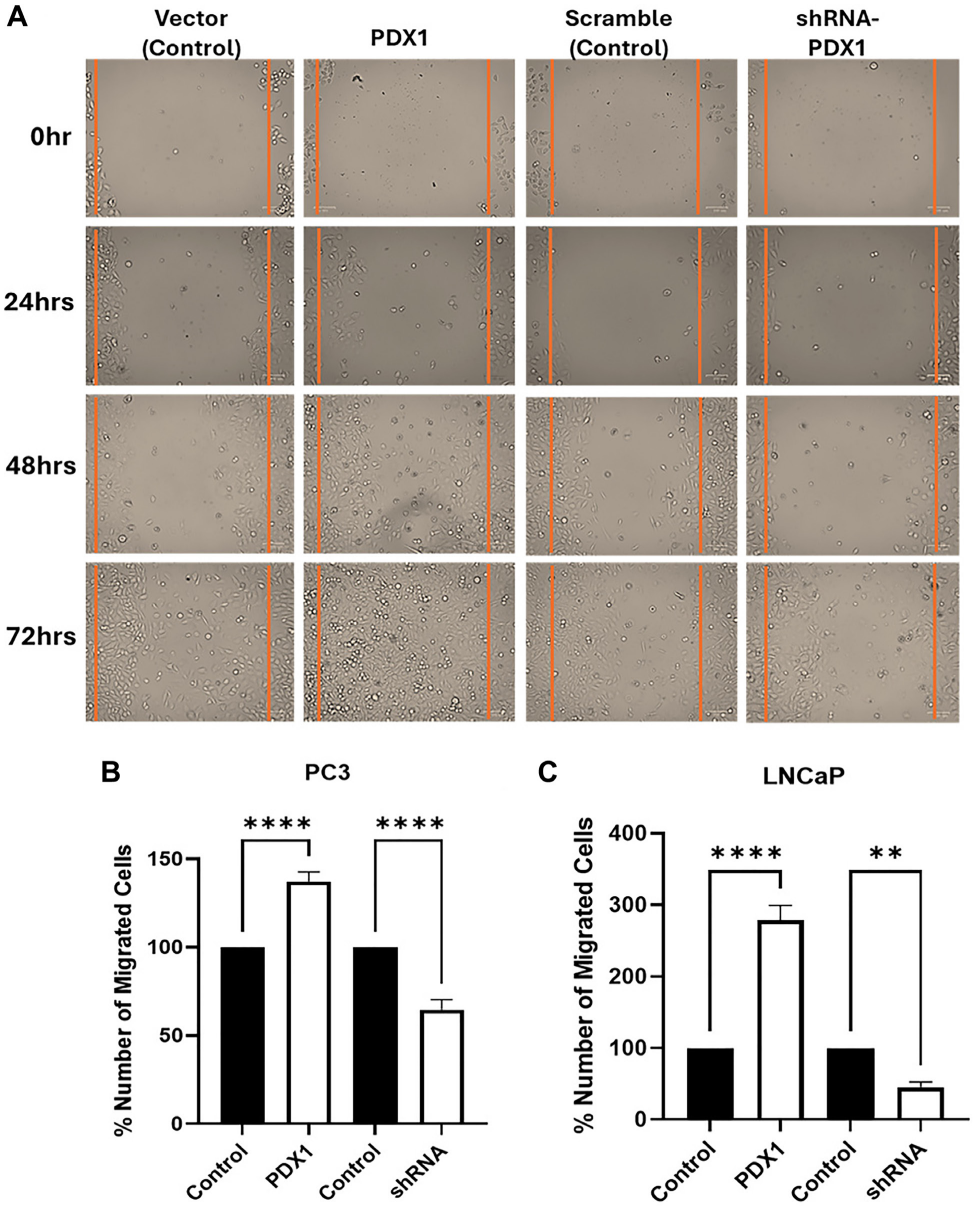
PDX1 expression and prostate cancer cell migration analysis. (**A**) PC-3 cells and LNCaP cells stably transfected with PDX1 expression vector or control only were used in scratch wound assay as described in materials and methods. The cells were permitted to migrate to the area of clearing for a total of 72 hrs and photomicrographs taken at 0, 24, 48 and 72 hrs. Results shown are typical of 3 separate experiments. (**B**) Quantitative analysis of transwell migration assay was used to evaluate the relative % number of migrated cells inPDX1 expressing cells or PDX1 shRNA knockdown cells compared to their respective controls in PC3. (**C**) Quantitative analysis of transwell migration assay was used to evaluate the relative % number of migrated cells in PDX1 overexpressing cells or PDX1 shRNA knockdown cells in LNCaP cells compared to their respective controls. Controls are set to 100% in (B) and (C). Data shown are representative of three independent experiments. Statistical significance is indicated as (^*^p < 0.05; *t*-test).

To assess the impact of PDX1 overexpression or knockdown on PC-3 cell migration, we employed a scratch wound assay to evaluate the rate of wound closure after scraping cells from a monolayer culture (Figure 3A). Confluent PC-3 cells were scraped, and migration was monitored over a 72-hour period. As shown in Figure 3A, control cells (neomycin-resistant but not overexpressing PDX1 through pCMV-vector transfection) exhibited slower migration and wound closure compared to cells overexpressing PDX1, which demonstrated significantly higher closure rates at both 48-hr and 72-hr time points. Similarly, scrambled shRNA cells (puromycin-resistant,) showed enhanced migration at the 48-hr and 72-hr time points compared to PDX1 shRNA knockdown cells. In support of these findings, transwell migration and invasion assays revealed that PDX1 overexpression significantly increased migration in both PC-3 and LNCaP cells compared to controls (*p* < 0.001; Figure 3B, 3C, respectively). In contrast, PDX1 knockdown via shRNA markedly reduced migration and invasion rates in both PC-3 and LNCaP cells compared to scrambled shRNA controls (*p* < 0.01; Figure 3B, 3C, respectively), with a more pronounced effect observed in PC-3 cells (*p* < 0.0001) compared to LNCaP cells. This experiment was conducted in triplicate, consistently yielding similar results. These findings underscore the critical role of PDX1 in promoting the migratory and invasive behaviors of prostate cancer cells.

### PDX1 regulation of signal targets in prostate cancer

**Figure 4:**
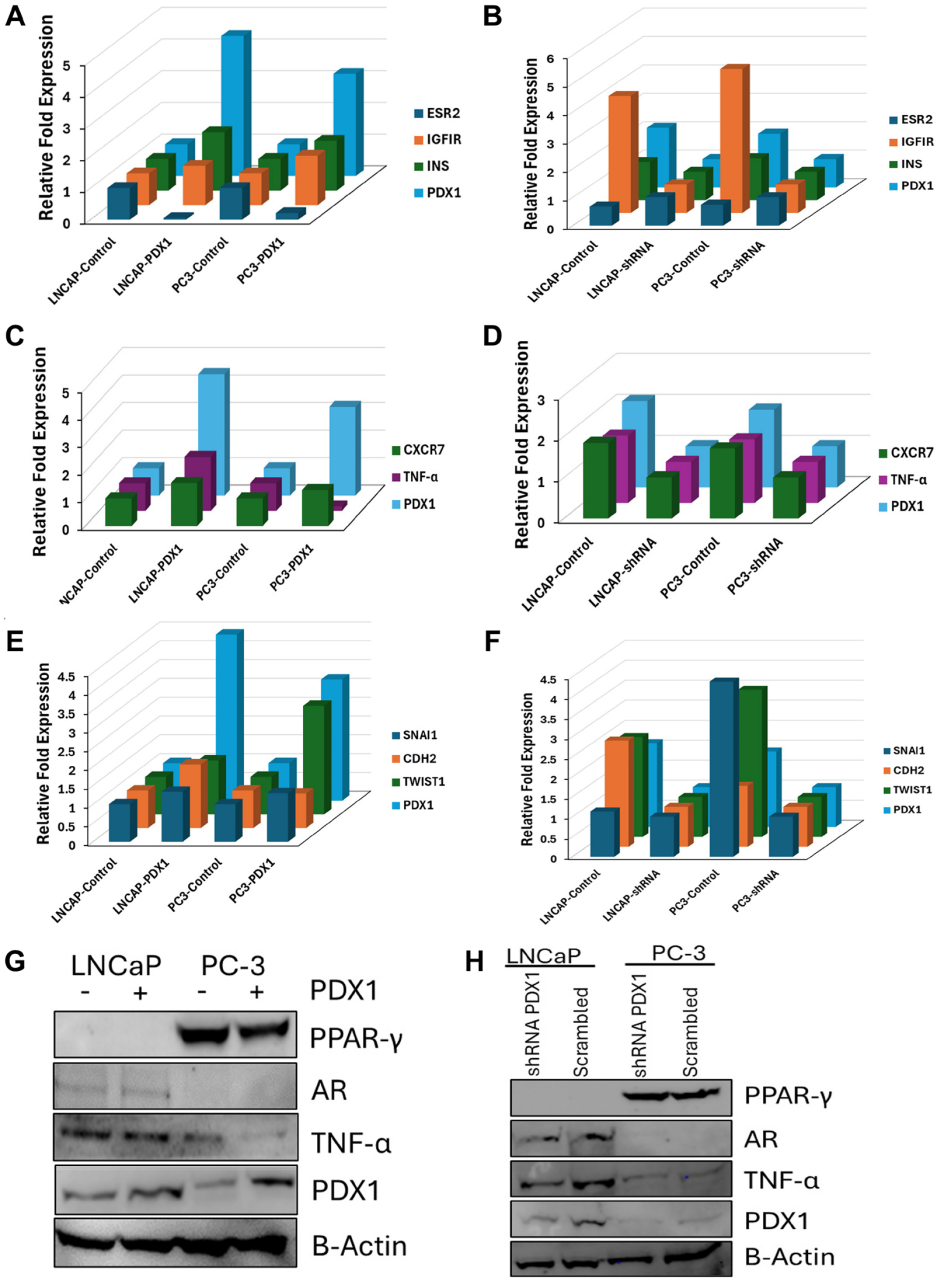
PDX1 modulates intermediates in insulin signaling, inflammatory, and EMT pathways in prostate cancer cells. (**A**) Relative qRT-PCR analysis of IGF1R, INS, and ESR2 transcript expression level in LNCaP and PC-3 cells engineered to stably express PDX1 compared to control. (**B**) Relative qRT-PCR analysis of IGF1R, INSR and ESR2 transcript expression level in LNCaP and PC-3 cells engineered with stable shRNA PDX1 knockdown compared to scramble shRNA control. (**C**) Relative qRT-PCR analysis of CXCR7, and TNFα transcript expression level in LNCaP and PC-3 cells engineered to stably express PDX1 compared to control transfection. (**D**) Relative qRT-PCR analysis of CXCR7, and TNFα in LNCaP and PC-3 cells engineered with stable PDX1 shRNA knockdown compared to control transfection. (**E**) Relative qRT-PCR analysis of SNAI1, CDH2, and TWIST1 transcript expression level in LNCaP and PC-3 cells engineered to stably express PDX1 compared to control transfection. (**F**) Relative qRT-PCR analysis of SNAI1, CDH2, and TWIST1 in LNCaP and PC-3 cells engineered with stable PDX1 shRNA knockdown compared to control transfection. Controls are set to 1 for (A–F). (**G**) Western blot analysis of PPAR-γ, AR, and TNFα, genes in LNCaP and PC-3 cells engineered to stably express PDX1 compared to control. (**H**) Western blot analysis of PPAR-γ, AR, and TNFα, genes in LNCaP and PC-3 cells with stable shRNA PDX1 knockdown compared to scramble control. Western blot bands quantification using ImageJ, and relative protein expression levels were normalized to β-actin in PDX1-overexpressing LNCaP and PC-3 cells. Data shown are representative of three independent experiments.

Diverse functions of PDX1, indispensable for pancreatic development and progression from normal exocrine cells to metastatic pancreatic ductal adenocarcinoma includes insulin signaling pathway, inflammatory pathways and epithelial-t-mesenchymal transition [34]. We aimed to investigate whether PDX1 affects the expression levels of several key intermediates in insulin signaling and prostate carcinogenesis using qRT-PCR and Western blot analysis in LNCaP and PC-3 cell lines. Quantitative RT-PCR (qRT-PCR) analysis expressed as relative fold change in expression of LNCaP cells overexpressing PDX1 (4.42-fold) revealed a modest increase in insulin-related markers, including IGF1R (1.24-fold) and INSR (1.84-fold), along with a significant reduction in ESR2 expression (19.32-fold) compared to control transfections (Figure 4A). Similarly, qRT-PCR analysis of PC-3 cells overexpressing PDX1 (3.22-fold) showed a modest increase in IGF1R (1.56-fold) and INSR (1.57-fold), with a significant reduction in ESR2 expression (4.81-fold) compared to control transfections (Figure 4A). In contrast, qRT-PCR analysis of LNCaP cells with PDX1 shRNA knockdown showed 2.22-fold decrease expression was associated with a significant reduction in IGF1R (4.11-fold) and INSR (1.3-fold), while ESR2 expression increased (1.49-fold) compared to scramble shRNA (control) transfections (Figure 4B). In PC-3 cells, PDX1 shRNA knockdown resulted in 1.89-fold reduction in expression, and this was associated with a significant reduction in IGF1R (5-fold) and INSR (1.47-fold), alongside an increase in ESR2 expression (1.36-fold) compared to control transfections (Figure 4B).

In addition, we investigated the role of PDX1 expression and inflammatory signaling in prostate carcinogenesis. In LNCaP cells overexpressing PDX1 (4.42-fold) exhibited modest increased expression of the inflammatory cytokines CXCR7 (1.56-fold) and TNF-α (1.95-fold), compared to control transfections (Figure 4C). In PC-3 cells, PDX1 overexpression (3.22-fold) resulted in a modest increased in CXCR7 expression (1.31-fold), but reduced expression of TNFα (6.25-fold) compared to the control transfection (Figure 4C). In contrast, PDX1 shRNA knockdown expression (0.48 -fold) in LNCaP cells was associated with a decrease expression in CXCR7 (1.84-fold), and TNFα (1.64-fold) compared to control transfected cells (Figure 4D). Similarly, PDX1 shRNA knockdown expression (0.53 -fold) in PC-3 cells resulted in a reduction in the expression of inflammatory markers CXCR7 (1.71-fold), and TNFα (1.55-fold) compared to control transfection (Figure 4D). We next examined the effect of PDX1 expression on epithelial-mesenchymal transition (EMT) markers through qRT-PCR analysis. In LNCaP cells, PDX1 overexpression (4.42-fold) resulted in modest increased expression of SNAI1 (1.33-fold), CDH2 (1.69-fold), and TWIST1 (1.43-fold) compared to the control transfection (Figure 4E). In PC-3 cells, overexpression of PDX1 (3.22-fold) enhanced SNAI1 (1.29-fold) and TWIST1 (2.89-fold) expression, while there was no significant change in CDH2 expression compared to control transfection (Figure 4E). In both LNCaP and PC-3 cell lines, PDX1 shRNA knockdown expression (0.48-fold, and 0.53-fold respectively) showed significant reduction in the expression of SNAI1, CDH2, and TWIST1 (>1.5-fold), except in LNCaP cells where the reduction in SNAI1 (1.33-fold) was modest compared to the control transfection (Figure 4F). Western blot analysis of selected proteins showed no significant changes in AR and TNF-α expression in LNCaP cells overexpressing PDX1 (Figure 4G) or LNCaP cells with PDX1 knockdown (Figure 4H). The PC3 cells over-expressing PDX1 showed reduction in TNF-α expression but no detectable difference in PPAR-γ expression (Figure 4G). Similarly, PC3 cells with PDX1 knockdown did not show detectable differences in PPAR-γ or TNF-α expression (Figure 4H). Overall, the qRT-PCR and Western blot data highlights critical roles of PDX1 expression in prostate carcinogenesis through modulating key regulatory intermediates.

### Molecular insights into glucose-dependent PDX1 activity and metabolic markers

**Figure 5:**
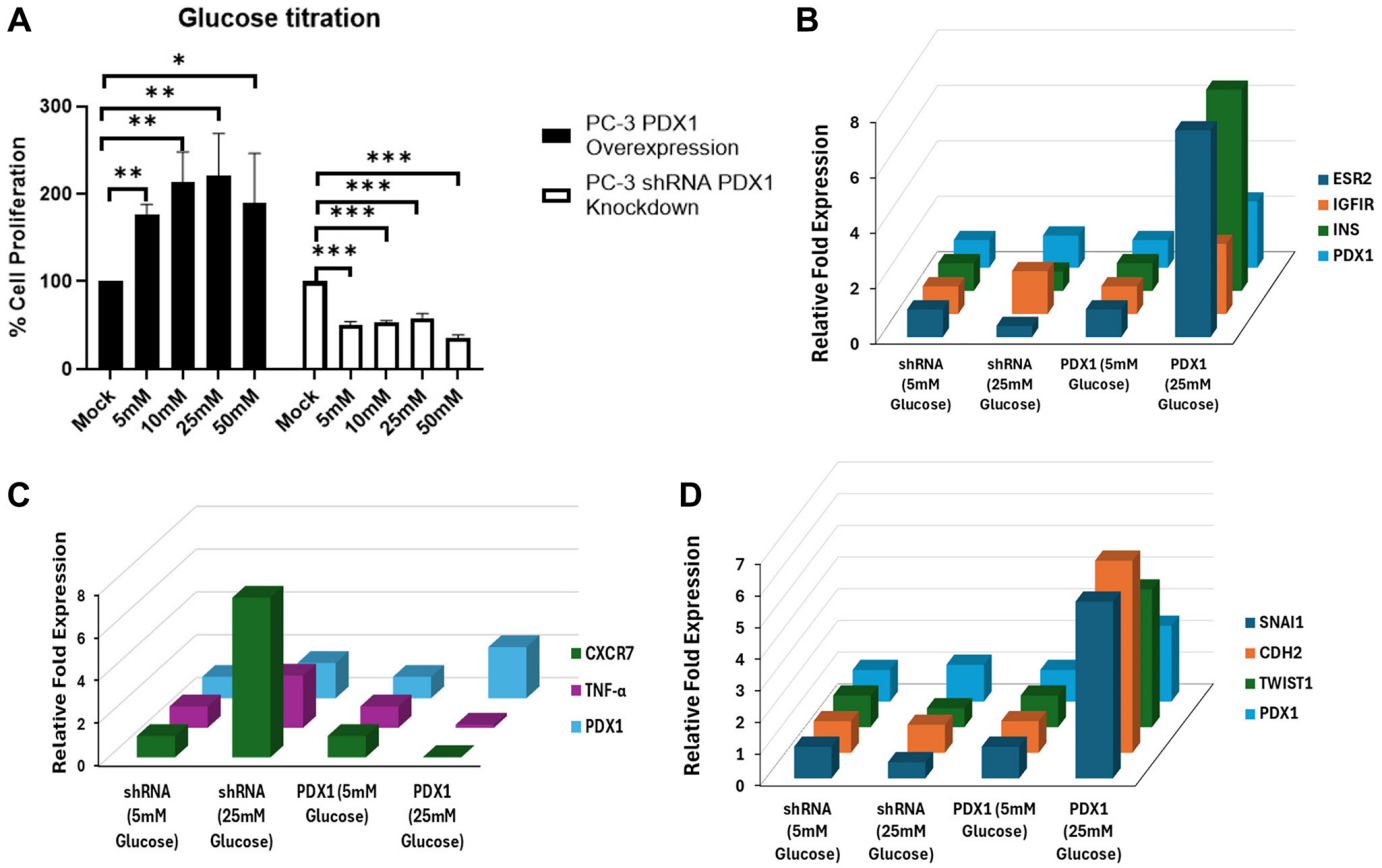
Analysis of glucose-dependent PDX1 activity and metabolic markers in prostate cancer cells. (**A**) Cell proliferation assay evaluating the effect of varying glucose concentrations (5 mM, 10 mM, 25 mM, and 50 mM; or no glucose- mock) in PC-3 cells stably expressing PDX1 overexpression or with stable shRNA PDX1 knockdown. Cells were counted at 24, 48, and 72 hrs post-treatment. Mock treated cells are set to 100%. (**B**) Relative qRT-PCR analysis of ESR2, IGF1R, and INSR in response to high glucose treatment compared to low glucose in PC3 cells with stable PDX1 expression or stable shRNA PDX1 knockdown compared to respective controls. (**C**) Relative qRT-PCR analysis of CXCR7, and TNFα in response to high glucose treatment compared to low glucose in PC3 with stable PDX1 expression or stable shRNA PDX1 knockdown compared to respective controls. (**D**) Relative qRT-PCR analysis of SNAI1, CDH2, and TWIST1 in response to high glucose treatment compared to low glucose in PC3 cells with stable PDX1 expression or stable shRNA PDX1 knockdown compared to respective controls. Controls are set to 1 for (**B**–D). Data shown are representative of three independent experiments.

The transcription factor PDX1 plays a critical role in glucose homeostasis, prompting us to investigate its expression in response to varying glucose concentrations. As shown in Figure 5A, PC-3 cells overexpressing PDX1 exhibited a significant, dose-dependent increase in cell proliferation when exposed to glucose concentrations ranging from 5 mM to 50 mM. The highest cell proliferation (2.22-fold) was observed at a 25 mM glucose concentration. In contrast, PC-3 cells with stable shRNA knockdown of PDX1 showed significant inhibition of cell proliferation across all glucose concentrations, with the highest inhibition (2.79-fold) observed at 50 mM glucose (Figure 5A).

We examined the expression of various metabolic markers in PC-3 cells stably overexpressing PDX1 or with shRNA-mediated PDX1 knockdown, following stimulation with either high glucose (25 mM) or low glucose (5 mM) (Figure 5B–5D). In PC-3 cells overexpressing PDX1, stimulation with high glucose led to significant increases in glucose-related markers, including ESR2 (7.47-fold), IGF1R (2.53-fold), INSR (7.26-fold), and PDX1 itself (2.39-fold) compared to low glucose stimulation (Figure 5B). Conversely, in PC-3 cells with stable PDX1 knockdown, there was a marked reduction in ESR2 (2.48-fold), and modest changes in INSR (1.43-fold), IGF1R (1.54-fold), and PDX1 (1.45-fold) expression following high glucose stimulation compared to low glucose (Figure 5B). Remarkable changes were observed in the expression of proinflammatory markers in response to glucose stimulation and PDX1 expression (Figure 5C). In PC-3 cells overexpressing PDX1, high glucose stimulation resulted in significant reductions in CXCR7 expression (55.7-fold), and TNFα expression (6.5-fold) compared to low glucose level. On the other hand, PC-3 cells with PDX1 knockdown showed significant increases inCXCR7 (7.46-fold), and TNFα (2.44-fold) expression in response to high glucose stimulation compared to low glucose (Figure 5C).

Finally, we assessed the expression of epithelial-mesenchymal transition (EMT) markers. In PC-3 cells overexpressing PDX1, high glucose stimulation resulted in significantly elevated levels of SNAI1 (5.58-fold), CDH2 (6.06-fold), and TWIST1 (4.35-fold) compared to low glucose stimulation (Figure 5D). In contrast, PC-3 cells with stable PDX1 knockdown showed only modest reductions in EMT marker expression in response to high glucose (Figure 5D). Overall, our findings underscore the important role of PDX1 in regulating various metabolic markers in response to glucose stimulation.

## DISCUSSION

Recent studies suggest that epigenetic dysregulation plays a key role in regulating the PDX1 expression in gastric cancer [35] and colorectal cancer [36]. DNA methylation signatures were shown to stratify tumors by subtype, highlighting PDX1 as a potential methylation biomarker for distinguishing high-risk patients from those with indolent disease in clinical practice. In our genome-wide DNA methylation analysis [28, 32] and the TCGA [33] datasets revealed that PDX1 is hypermethylated in prostate tumors compared to benign tissues, with methylation frequency significantly correlating with Gleason Score. In the present study, we found significant increased expression of PDX1 in PCa compared to adjacent matched normal tissues consistent with the TCGA dataset. In addition, Jonmaker et al. [37] have reported PDX1 overexpression in prostate tumors versus benign tissues based on immunohistochemistry analysis of a tissue microarray. We report in this study, hypermethylation spanning the PDX1 promoter and gene body (canyon). The positive correlation between PDX1 hypermethylation and expression in PCa as reported by us and the TCGA dataset, can be explained by previous reports that demonstrates that gene body hypermethylation, but not promoter can directly increase its gene expression [38]. Indeed, studies carried out by Su et al. [38] have demonstrated that hypermethylated canyons are enriched for homeobox genes and oncogenes. They noted that about 67% of homeobox genes are associated with reference canyons and suggest that gene-body canyon hypermethylation might be a dominant epigenetic mechanism for homeobox oncogene activation in cancer.

PDX1 demonstrates oncogenic activity, as its overexpression in pancreatic ductal adenocarcinoma (PDA) cell lines promote proliferation, invasiveness, and anchorage-independent growth [39]. However, large scale studies suggest that PDX1 loss is associated with a more aggressive PDA subtype [40]. This apparent paradox is attributed to PDX1 undergoing functional transitions during pancreatic oncogenesis: (i) PDX1 initially maintains acinar cell identity and prevents pancreatic intraepithelial neoplasia (PanIN), the precursor to PDAC, functioning as a tumor suppressor at this stage [34]; (ii) during neoplastic transformation, PDX1 shifts to an oncogenic role, driving proliferation and inhibiting apoptosis; and (iii) during epithelial–mesenchymal transition (EMT), PDX1 expression diminishes, facilitating metastasis, thus acting as an EMT suppressor [34]. In other cancers, PDX1 has been proposed as a tumor suppressor, including gastric cancer [41] and breast cancer [42]. However, its role in prostate cancer remains poorly understood. In this study, we demonstrated that PDX1 regulates cell proliferation and invasion *in vitro*, with PDX1 overexpression significantly enhancing proliferation, and migration in human PCa cells. Furthermore, PDX1 suppression via shRNA reversed these effects, suggesting an oncogenic role for PDX1 in prostate cancer.

Multiple studies have demonstrated that the insulin/Insulin-like Growth Factor 1 (INS/IGF-1) axis plays a crucial role in driving tumor growth. Insulin-like growth factor binding proteins (IGFBPs) regulate the bioavailability of IGF-1. Overexpression of PDX1 leads to increased expression of INSR and IGF-1/IGFBP signaling [43]. However, the relationship between serum IGF-1 levels and the risk of developing prostate cancer (PCa) remains unclear, with some studies indicating an increased risk or no significant correlation [44]. Our findings reveal a notable crosstalk and a positive correlation between PDX1 and INSR as well as IGFBP1 expression, particularly under conditions of high glucose in PCa cells. It is well established that androgen mediates prostate growth and development through Androgen Receptor (AR), which is a principal therapeutic target for androgen therapy in prostate cancer [45]. Androgen receptor has been shown to increase glucose uptake in LNCaP cells [46]. We did not observe any significant effect of PDX1 in AR expression in LNCaP cells, suggesting these are separate pathways in regulating glucose signaling. Estrogen has also been shown to be important in prostate carcinogenesis [47]. The prostate expresses both estrogen receptor alpha (ERα) and estrogen receptor beta (ERβ) and most in-vitro cancer cell lines, animal and human cancer studies suggest that ERα mediates oncogenic effects of estrogen in the prostate, whereas ERβ has more tumor suppressive function [48]. The ERβ is encoded by *ESR2* gene and ERβ is implicated in the regulation of glucose homeostasis and insulin signaling as well as inflammatory pathways [49]. A previous study found that estrogen receptor (ERα) binds and repress the transcriptional activity of PDX1 and insulin expression in hamster insulinoma HIT-T15 cells [50]. We have observed an inverse correlation between PDX1 and ESR2 expressions. However, in PC3 cells stably expressing PDX1, exposure to high glucose significantly increased ESR2 expression, suggesting that high glucose concentration was able to override the PDX1-mediated inhibitory effects of ESR2 expression. This observation indicates that ESR2 may regulate insulin transcription by indirect genomic signaling involving PDX1 transcription. Peroxisome proliferator-activated receptor gamma (PPAR-γ) regulates genes important in glucose homeostasis [51] and a previous study suggests that PPAR-γ regulates pancreatic beta cell function by improving the stability of PDX1 protein [52]. We observed very high expression of PPAR-γ in PC3 cells but no significant difference in PDX1 expression cells and control. On the other hand, there was no detectable expression of PPAR-γ in LNCaP cells as it has been reported that PPAR-γ is transcriptionally inactive [53].

The activation of the epithelial-to-mesenchymal transition (EMT) program is a key driver of tumor progression, from initiation to metastasis. A study using an inducible PDX1 pancreatic cancer model found that depletion of the EMT regulator Zeb1—a critical factor in precursor lesion formation, invasion, and metastasis—suppressed stemness, colonization capacity, and notably, the phenotypic and metabolic plasticity of pancreatic tumor cells [54]. Our findings reveal a positive correlation between PDX1 expression and EMT markers, including SNAI1, CDH2, and TWIST1, suggesting that PDX1 oncogenic activity may be mediated through EMT activation, particularly under high-glucose conditions. The PDX1 protein has been reported to have anti-inflammatory effects in pancreatic beta cells by repressing nuclear factor kappa-light-chain-enhancer of activated B cells (NF-kB; [55]). However, other studies have reported pro-inflammatory role for tumor necrosis factor-alpha (TNF-α), an upstream activator of NF-kB. For instance, increased expression of TNF-α has been observed in prostate cancer (PCa) patients with metabolic syndrome (MS) complications compared to those without MS [56]. Another study reported TNF-α as a positive regulator of PDX1 gene promoter in pancreatic beta cells [57]. We have found significant reduced expression of TNF-α in PC3 cells expressing PDX1 by qRT-PCR and Western blot analysis, whereas we did not observe any significant differences in LNCaP cells expressing PDX1 suggesting the association between PDX1 and TNF-α is dependent on the cellular context. We found that elevated PDX1 expression was associated with increased levels of inflammatory chemokine, CXCR7, whereas PDX1 loss correlated with the reduced CXCR7 expression. Interestingly, in the absence of PDX1 expression, high glucose exposure caused increased CXCR7 expression whereas PDX1-expression in high glucose exposure suppressed CXCR7 expression, suggesting that while high-glucose conditions or PDX1 expression can enhance CXCR7 expression, the dual expression of PDX1 and high glucose can inhibit CXCR7 expression perhaps via multiple signaling pathways.

In summary, our findings highlight PDX1 as a key regulator of prostate cancer (PCa), playing a pivotal role in multiple signal transduction pathways critical for prostate carcinogenesis. Its influence is particularly pronounced under high-glucose conditions, which mimic the hyperglycemic state observed in PCa patients with diabetes. Given its master regulatory function in PCa, PDX1 may serve as a valuable marker for disease progression and a potential future therapeutic target, particularly for patients with co-morbid diabetes and/or obesity.

## MATERIALS AND METHODS

### Cell culture

The human study was approved by the Georgetown University Medical Center Institutional Review Board (IRB; # 1992-048, Washington DC) and the Howard University IRB (19-MED-08, Washington, DC). The human PCa cell lines; PC3 (androgen-independent) and LNCaP (androgen-dependent) were obtained from the ATCC (ATCC, Manassas, VA, USA). The cell lines were cultured in RPMI-1640 medium supplemented with 10% fetal bovine serum (Gibco, Thermo Fisher Scientific, Waltham, MA, USA) and 1% Penicillin streptomycin (Cellgro, Corning, Thermo Fisher Scientific, Waltham, MA, USA), unless stated otherwise. All cell lines were maintained at 37°C in a humidified atmosphere of 5% CO_2_.

### Cell transfection assay

For PDX1 overexpression, the LNCaP and PC3 cells were seeded at a density of 0.5 × 10^5^ cells/well in a 12-well plate. After 24-h incubation period, the cells were transfected with 1.5, 2, or 2.5 μg/well of the PDX1 plasmid construct pCMV-PDX1 (Myc-DDK-tagged) (OriGene- Cat. No. - RC222354) or an empty pCMV vector (Origene, Rockville, MD, USA) using Lipofectamine LTX transfection reagent (Invitrogen, Waltham, MA, USA) following the manufacturer protocol. Cells were trypsinized and counted using a Coulter counter at 24, 48, or 72 hours after transfection. To establish stably transfected cells, cells were selected in neomycin (Gibco) containing medium at a final concentration of 400 μg/ml the day after post transfection. Three weeks following selection with neomycin, the neomycin-resistant clones were pooled and propagated. The propagated transfected cells were subsequently used for RNA and protein extraction to perform quantitative RT-PCR, Western blot analysis, and other relevant analyses. For PDX1 Knockdown, LNCaP and PC-3 cells were seeded at a density of 1 × 10^6^ cells per well in T25 flasks and transfected with either the PDX1-targeting shRNA or scrambled negative control shRNA (ABM biolabs, Vancouver, CN; Cat No. LV015-G-Custom) using DNAfectin Plus transfection reagent (ABM Cat No. G2500), following the manufacturer’s protocol. After 24 hours, the transfected cells were cultured in a medium containing puromycin (Thermo Fisher Scientific Cat No. J67236) at a final concentration of 3 μg/ml for selection. Cells were trypsinized and counted using a Coulter counter at 24- and 48-hrs post-selection. At 48 hrs, one plate of transfected cells was used for RNA extraction to assess PDX1 knockdown efficiency through qRT-PCR. The shRNA construct that demonstrated the most effective growth inhibition and greatest PDX1 suppression was identified and combined into a shRNA duplex. Puromycin-resistant clones were pooled and propagated over a two-week period. The resulting stably transfected cell lines were used for RNA and protein extraction to perform quantitative RT-PCR, Western blot analysis, and other downstream assays.

### Cell proliferation assays

For gain-or-loss of PDX1 expression analysis, cell proliferation was assessed using a cell-counting kit from Bio-Rad. Cells were seeded in 12-well plates at a density of 1.5 × 10^5^ cells per well and cultured under standard conditions. At predetermined time points (24, 48, and 72 hours), 10 μL of the cell suspension was combined with 10 μL of 0.4% trypan blue staining solution. The mixture was then analyzed using the TC20 Automated Cell Counter (Bio-Rad, Hercules, CA, USA) to distinguish between live and dead cells. All experiments were performed in triplicate to ensure accuracy and reproducibility.

### Quantitative real time-PCR (qRT-PCR) analyses

Total RNA was extracted from cells or tissues using TRIzol reagent (Invitrogen, Cat No. 15596026) and subsequently reverse-transcribed into cDNA with the RevertAid RT kit (ThermoScientific, Cat No. K1691) following the manufacturer’s instructions. The resulting cDNA served as the template for reverse transcription quantitative PCR (RT-qPCR) to analyze gene expression. TaqMan assays (Table 1) were performed using HotStarTaq Master Mix (Qiagen, Cat No. 203203) on a CFX96 Real-Time PCR System (Bio-Rad, Hercules, CA, USA). The thermal cycling protocol included an initial heat activation at 95°C for 15 minutes, followed by 35 cycles of 94°C for 30 seconds, 54°C for 30 seconds, and 72°C for 1 minute. Gene expression fold changes were calculated using the 2^–ΔΔCt^ method, with β-actin serving as the endogenous control. All RT-qPCR experiments were conducted in triplicate to ensure reliability and reproducibility.

**Table 1:** Taqman primer oligonucleotide sequence information used in quantitative RT-PCR analysis

Gene abbreviations	Forward primer (5′–3′)	Reverse primer (5′–3′)	Taqman (5′–3′)
β-actin	GAACTGCCTGACTACCTCATG	CGAAGTCCAGGGCAACATAG	TGCGTGACATCAAAGAGAAGCTGTGC
CDH2	GTTTGCCAGTGTGACTCCA	CATACCACAAACATCAGCACAAG	TCATTGCCATCCTGCTCTGCATCA
CXCR7	GCTCACAGTTGTTGCAAAGTG	GAAGAGATGCAGATCCATCGT	AATCAAATGACCTCCGGGCTGGC
ESR2	CACTTCATGTTGAGCAGATGTTC	TCTCCTCCCAGCAGCAAT	CCTTGTTACTCGCATGCCTGACGT
GAPDH	GGTGTGAACCATGAGAAGTATGA	GAGTCCTTCCACGATACCAAAG	AGATCATCAGCAATGCC TCCTGCA
1GF1R	CTTATTGGCGTTGAGGTATGC	AGTTATCTCCGGTCTCTGAGG	TCATCTTGCTCAGGCTTGGAGGTG
INSR	TCTGATTCGAGGAGAGACCTT	AGGTTGTGTTTGCTCCAGTC	AGCTGCCTTAGGTTCTGGTTGTCC
PDX1	TGAAGTCTACCAAAGCTCACG	TCCTTCTCCAGCTCTAGCA	CCTGCCCACTGGCCTTTCCA
SNAIL1	GGCTGCTACAAGGCCAT	GCACTGGTACTTCTTGACATCT	TTCGCTGACCGCTCCAACCT
TNF-α	GAGACAGAAAGAGCGGGAAATA	ATTCACCTTCCAGGCATTCA	TTTCCCTGAGTGTCTTCTGTGTGCC
TWIST1	ATGTCCGCGTCCCATCA	ACTGTCCATTTTCTCCTTCTCTG	ATGACATCTAGGTCTCCGGCCCT

### Western blotting

Western blot analysis was performed following established protocols from our laboratory [58]. Protein samples were separated using NuPAGE 4–12% Bis-Tris gels and visualized with an enhanced chemiluminescence (ECL) detection system (Bio-Rad, Hercules, CA, USA). Imaging the membranes was carried out using a ChemiDoc MP imaging system (Bio-Rad). To ensure protein integrity, lysis buffers were supplemented with 0.5 M EDTA and a cocktail of protease and phosphatase inhibitors (Thermo Scientific, Cat No. 1861281) immediately prior to use. Protein concentrations were quantified using the BCA Protein Assay Kit (Pierce Inc., Rockford, IL, USA). All primary antibodies obtained from Santa Cruz Biotechnology (Dallas, TX) are as follows: NF-kB (1:500; sc-8008; Lot# H1220); C-myc (1:500; sc-40; Lot# J0220); AR (1:1000; sc-7305; Lot# J2920); TNFα (1:500; sc-515766 Lot# 17020); anti-PDX1 (Proteintech, Cat No. 20989-1-AP); and anti-PPAR-γ (Abcam, Cat No. ab178860). The β-actin antibody (1:500; sc-69879; Lot# D1911) was used as an internal loading control. Secondary antibodies, including anti-mouse and anti-rabbit antibodies, were purchased from Cell Signaling Technology (Cat No. 7076P2 and 7074P2, respectively).

### Wound healing and transwell invasion assays

A wound healing assay was carried out to evaluate cell migration. PC-3 and LNCaP cells were seeded at a density of 0.3 × 10^6^ cells per well in 6-well plates and cultured in an appropriate complete growth medium until reaching confluence. Using a sterile plastic pipette tip, a scratch was carefully introduced across the cell monolayer. The medium containing detached cells was removed, and each well was gently washed with PBS twice to remove debris. Fresh appropriate complete medium was then added to each well, and the cells were incubated to allow migration into the scratch area. Microscopic images of the wound area were obtained at 0, 24, 48, and 72 hrs to assess the progression of closure of the wound. Invasion assays were evaluated using transwell chambers (Sigma-Aldrich, Catalog No. ECM550) according to the manufacturer’s protocol.

### Tissue microarrays and immunohistochemistry

Archival Formalin-fixed paraffin-embedded (FFPE) tissue blocks of matched prostate adenocarcinoma and adjacent normal prostate tissues from 25 radical prostatectomy PCa patients collected from MedStar Georgetown University Hospital for surgical events in the period 2009–2015 were used in immunohistochemistry. Immunohistochemical staining of prostate cancer was performed for PDX-1 using rabbit recombinant monoclonal antibody. Five-micron sections from FFPE tissues were de-paraffinized with xylenes and rehydrated through a graded alcohol series. Heat induced epitope retrieval (HIER) was performed by immersing the tissue sections HighFlex pH (DAKO #K8004) in the PTLink (DAKO). Immunohistochemical staining was performed using a horseradish peroxidase labeled polymer from Dako (K4003) according to manufacturer’s instructions. Briefly, slides were treated with 3% hydrogen peroxide and 10% normal goat serum for 10 minutes each and exposed to primary antibodies for PDX-1 (1/100, Abcam #ab134150) for 1 hour at room temperature. Slides were exposed to the appropriate HRP labeled polymer for 30 min and DAB chromagen (Dako) for 5 minutes. Slides were counterstained with Hematoxylin (Fisher, Harris Modified Hematoxylin), blued in 1% ammonium hydroxide, dehydrated, and mounted with Acrymount. Consecutive sections with the primary antibody omitted were used as negative controls. Wash buffer used 1XTBS with 0.05% tween20 (Fisher).

The 25 prostate cancer slides were scanned using an Aperio GT450 Leica scanner. The immunostaining was semi-quantitatively analyzed based on the intensity of the expression of PDX-1 as well as its pattern of localization. This semi-quantitative scoring system, often on a scale like 0 to 3. Staining intensity was evaluated in the normal and prostate cancer epithelial cells as described previously. Staining intensity was graded as absent (0), weak (1+), intermediate (2+), or strong (3+). The extent of staining was estimated and scored as follows: 0% of cell no staining (0 score); 1–10% of cell stained (1 score); 11–50% of cell stained (2 score); or 51–80% of cells stained (3).

### Glucose titration assay

PC3 cell lines with stable PDX1 knockdown and overexpression were seeded in 12-well plates at a density of 1 × 10^5^ cells per well. The cells were cultured in their respective complete media under standard conditions. After overnight incubation, the complete media were replaced with glucose-free serum media supplemented with varying concentrations of glucose: 0 mM, 5 mM, 10 mM, 25 mM, and 50 mM (D-glucose; Sigma-Aldrich, Burlington, MA, USA). Cell viability was assessed by trypsinizing and counting the cells using a Coulter counter at 24, 48, and 72 hrs post-treatment. At the 24-hr mark, cells cultured with 0 mM glucose did not survive, indicating a critical dependency on glucose for cell viability. The optimal glucose concentration for subsequent experiments was determined to be 25 mM, which was used for qRT-PCR analysis to evaluate gene expression profiles in response to glucose treatment. A 5 mM glucose concentration served as the control for these analyses.

### Statistical analysis

All experiments were repeated three times, and results are presented as the mean ± SD. Analyses of significance were performed using Student’s *t*-tests, Fisher test or one-way ANOVA. *P* < 0.05 was considered statistically significant.

## Abbreviations

AR: Androgen Receptor
CDH2: Cadherin 2
CpG: Cytosine-phosphate-Cuanine
CXCR7: Chemokine (C-X-C Motif) Receptor 7
EMT: Epithelial to Mesenchymal Transition
ESR2: Estrogen Receptor 2
IGF-1: Insulin Like Growth Factor 1
IGF1R: Insulin Like Growth Factor 1 Receptor
INSR: Insulin Receptor
PDX1: Pancreatic And Duodenal Homeobox 1
NF-kB: Nuclear Factor Kappa B
PCa: Prostate Cancer
PPAR-γ: Peroxisome Proliferator Activated Receptor Gamma
TCGA: The Cancer Genome Atlas Program
TNFα: Tumor Necrosis Factor Alpha
SNAI1: Snail Family Transcriptional Repressor 1
TWIST: Twist Family BHLH Transcription Factor 1
